# Supplementary material for: Growing Maize Root: Lectins Involved in Consecutive Stages of Cell Development
Source: Plants (Basel). 2022 Jul 7;11(14):1799. doi: 10.3390/plants11141799 (PMC9319948; doi:10.3390/plants11141799)
Supplement: Supplementary file 1 [file plants-11-01799-s001.zip › Supplementary File 1 (S1-S7)(new).pdf]

Supplementary File (Figures S1-S7)

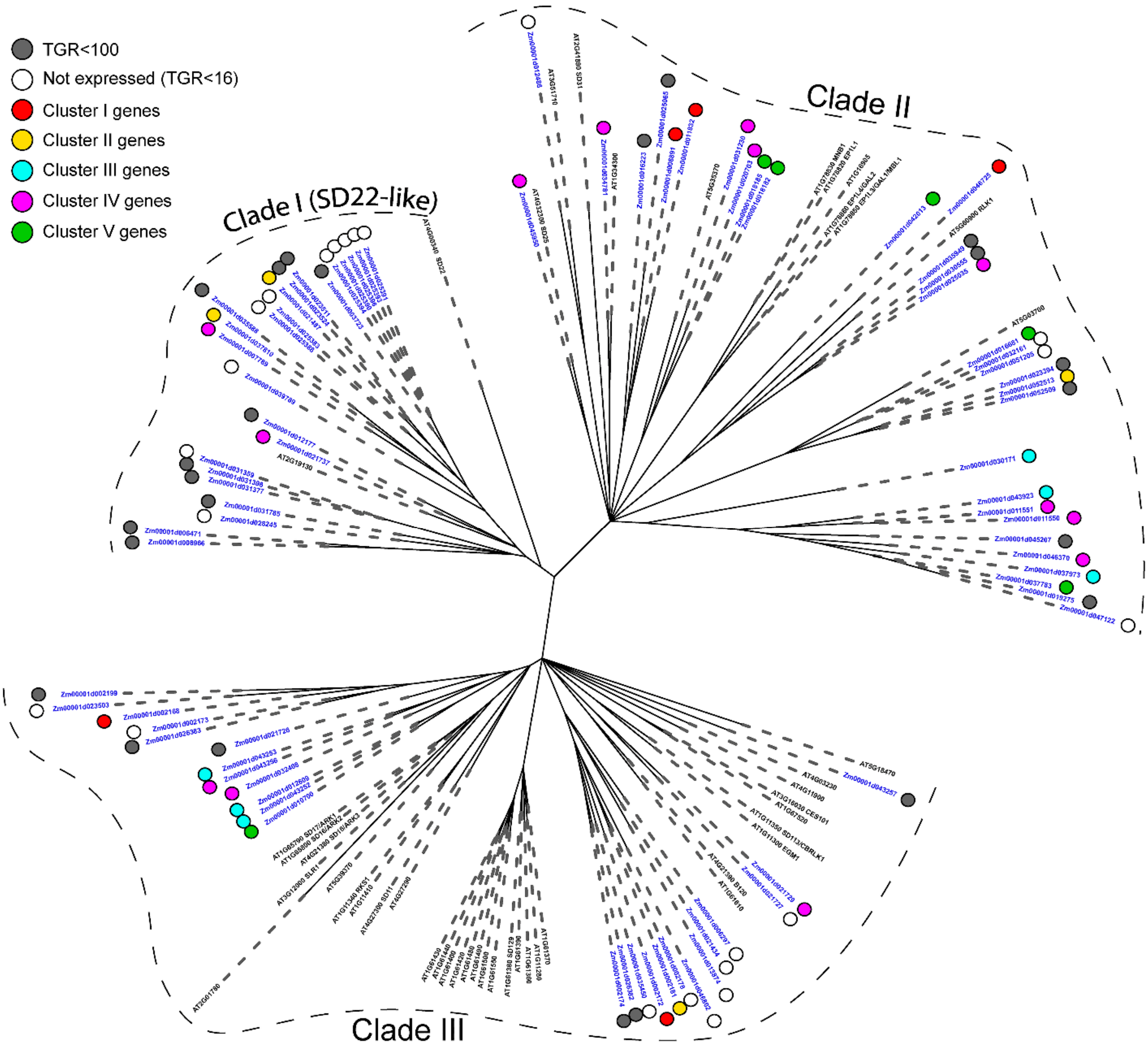

Figure S1. The phylogenetic dendrogram of *Galanthus nivalis* agglutinin (PF01453) family members of *A. thaliana* (gene names are given in black font) and *Z. mays* (gene names are given in blue font). The different colored dots next to *Z. mays* gene names indicate genes with different expression patterns: white are genes with no expression (TGR value less than 16 in all samples) in studied zones of maize root; grey are genes with TGR value less than 100; red are genes from I cluster, the expression decreased from the zones of meristem and root cap to the late elongation; yellow are genes from II cluster, the expression peak was at the initiation of cell elongation; blue are genes from III cluster, they showed a high expression from the initiation to late cell elongation; purple are genes from IV cluster, the expression increased in the root cap and also raised from the meristem zone to the late elongation; green are genes from V cluster, the expression raised to the late elongation. Only branches with the ultrafast bootstrap support values more than 90 are shown.

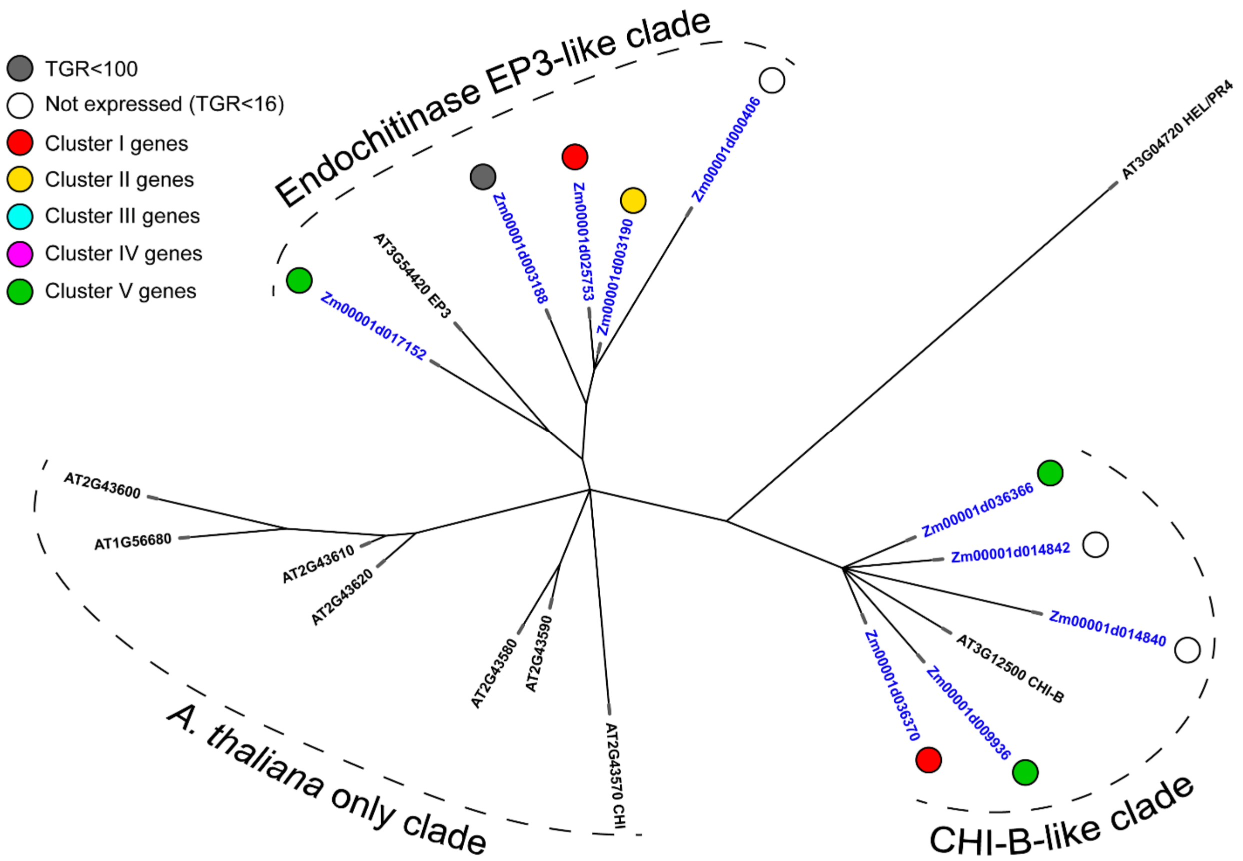

Figure S2. The phylogenetic dendrogram of hevein (PF00187) lectin family members of *A. thaliana* (gene names are given in black font) and *Z. mays* (gene names are given in blue font). The different colored dots next to *Z. mays* gene names indicate genes with different expression patterns: white are genes with no expression (TGR value less than 16 in all samples) in studied zones of maize root; grey are genes with TGR value less than 100; red are genes from I cluster, the expression decreased from the zones of meristem and root cap to the late elongation; yellow are genes from II cluster, the expression peak was at the initiation of cell elongation; blue are genes from III cluster, they showed a high expression from the start to the late elongation; purple are genes from IV cluster, the expression increased in the root cap and also raised from the meristem zone to the late elongation; green are genes from V cluster, the expression raised to the late elongation. Only branches with the ultrafast bootstrap support values more than 90 are shown.

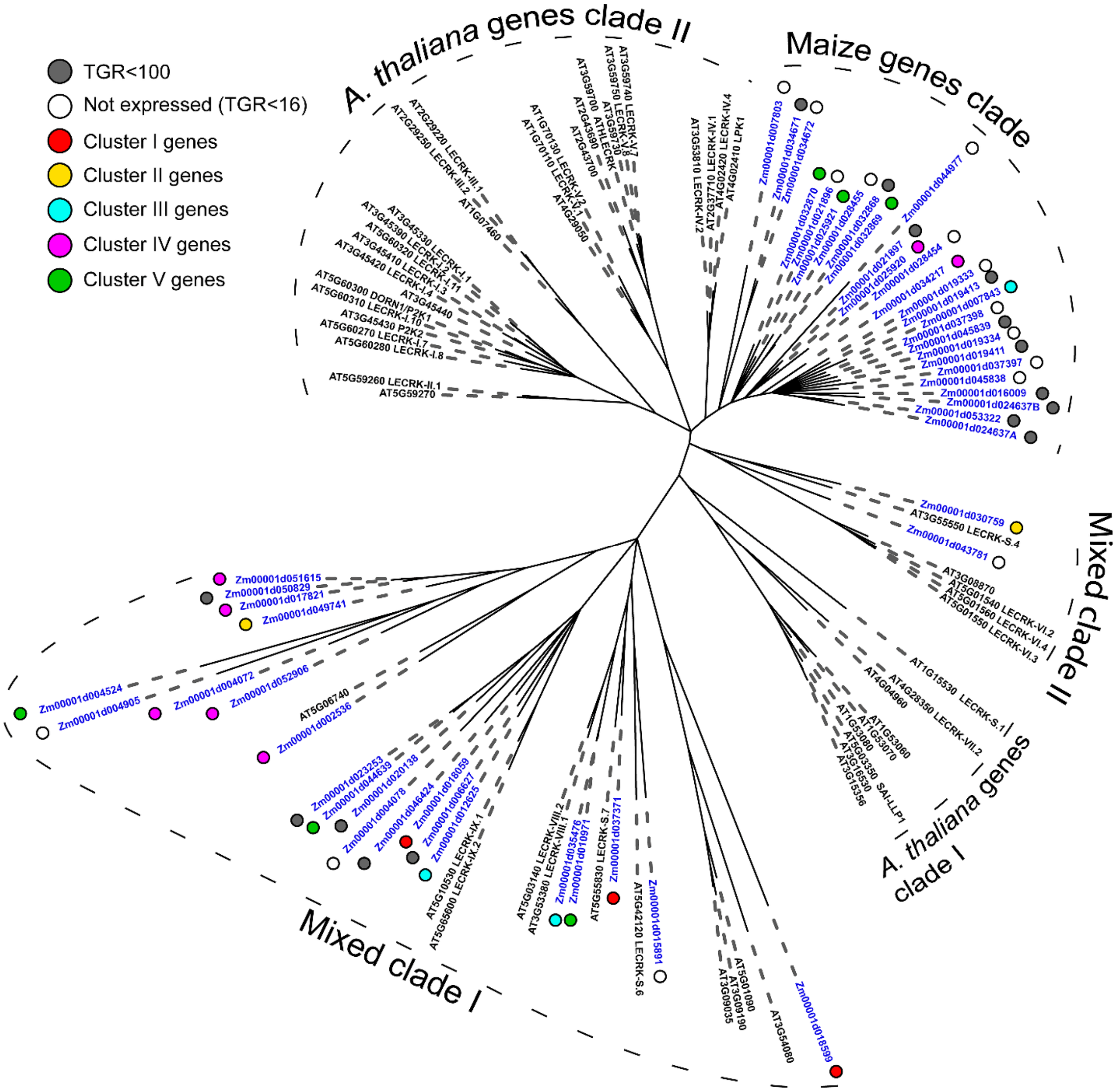

Figure S3. The phylogenetic dendrogram of legume (PF00139) lectin family members of *A. thaliana* (gene names are given in black font) and *Z. mays* (gene names are given in blue font). The different colored dots next to *Z. mays* gene names indicate genes with different expression patterns: white are genes with no expression (TGR value less than 16 in all samples) in studied zones of maize root; grey are genes with TGR value less than 100; red are genes from I cluster, the expression decreased from the zones of meristem and root cap to the late elongation; yellow are genes from II cluster, the expression peak was at the initiation of cell elongation; blue are genes from III cluster, they showed a high expression from the start to the late elongation; purple are genes from IV cluster, the expression increased in the root cap and also raised from the meristem zone to the late elongation; green are genes from V cluster, the expression raised to the late elongation. Only branches with the ultrafast bootstrap support values more than 90 are showed.

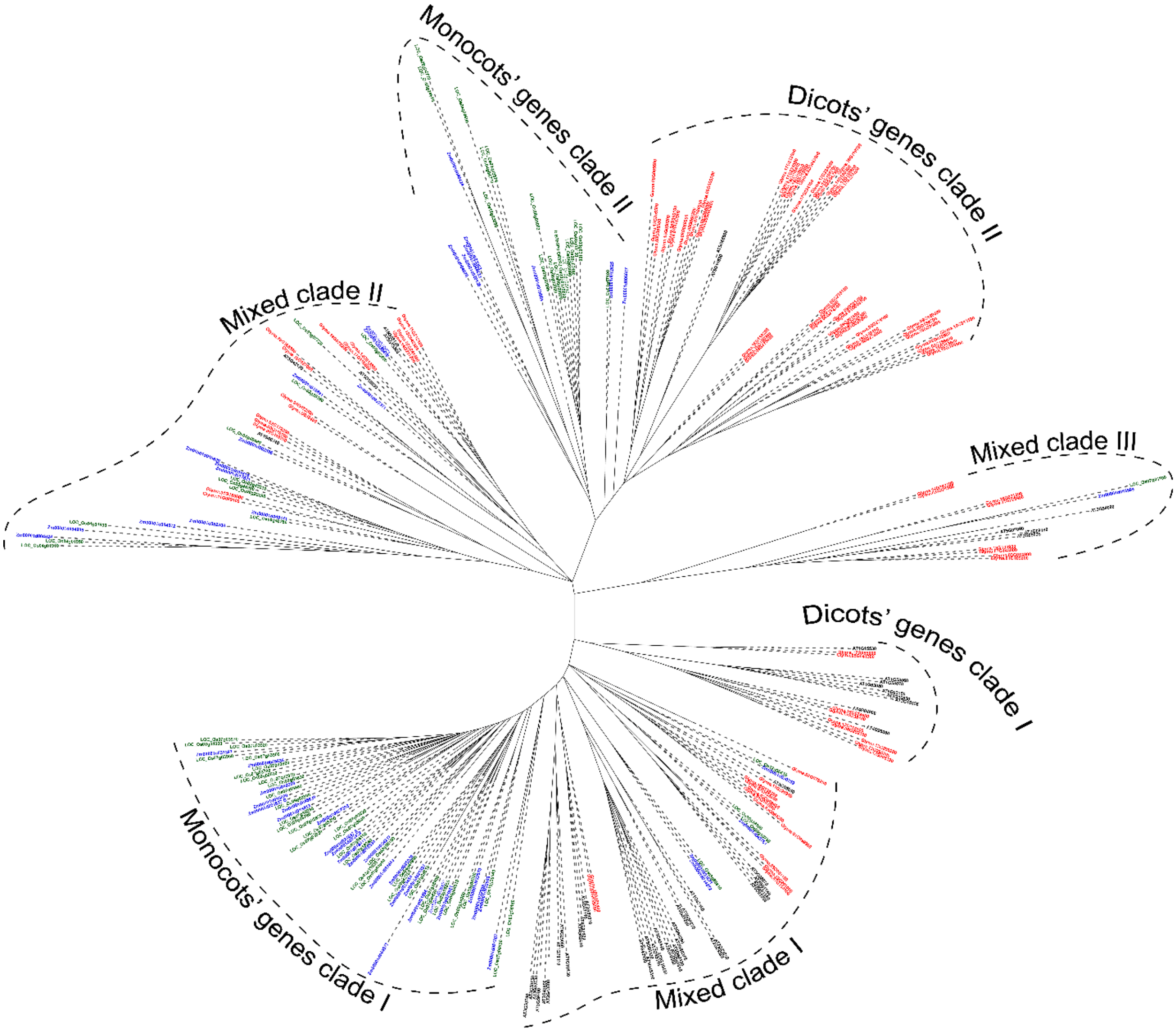

Figure S4. The phylogenetic dendrogram of legume (PF00139) lectin family members of *A. thaliana* (gene names are given in black font), *Z. mays* (gene names are given in blue font), *G. max* (gene names are given in red font) and *O. sativa* (gene names are given in green font). Only branches with the ultrafast bootstrap support values more than 70 are shown. Multiple alignment for phylogenetic analysis was provided using The Guidance2 server, and resulted Robinson-Foulds distance between ML tree and consensus tree was equal to 6.

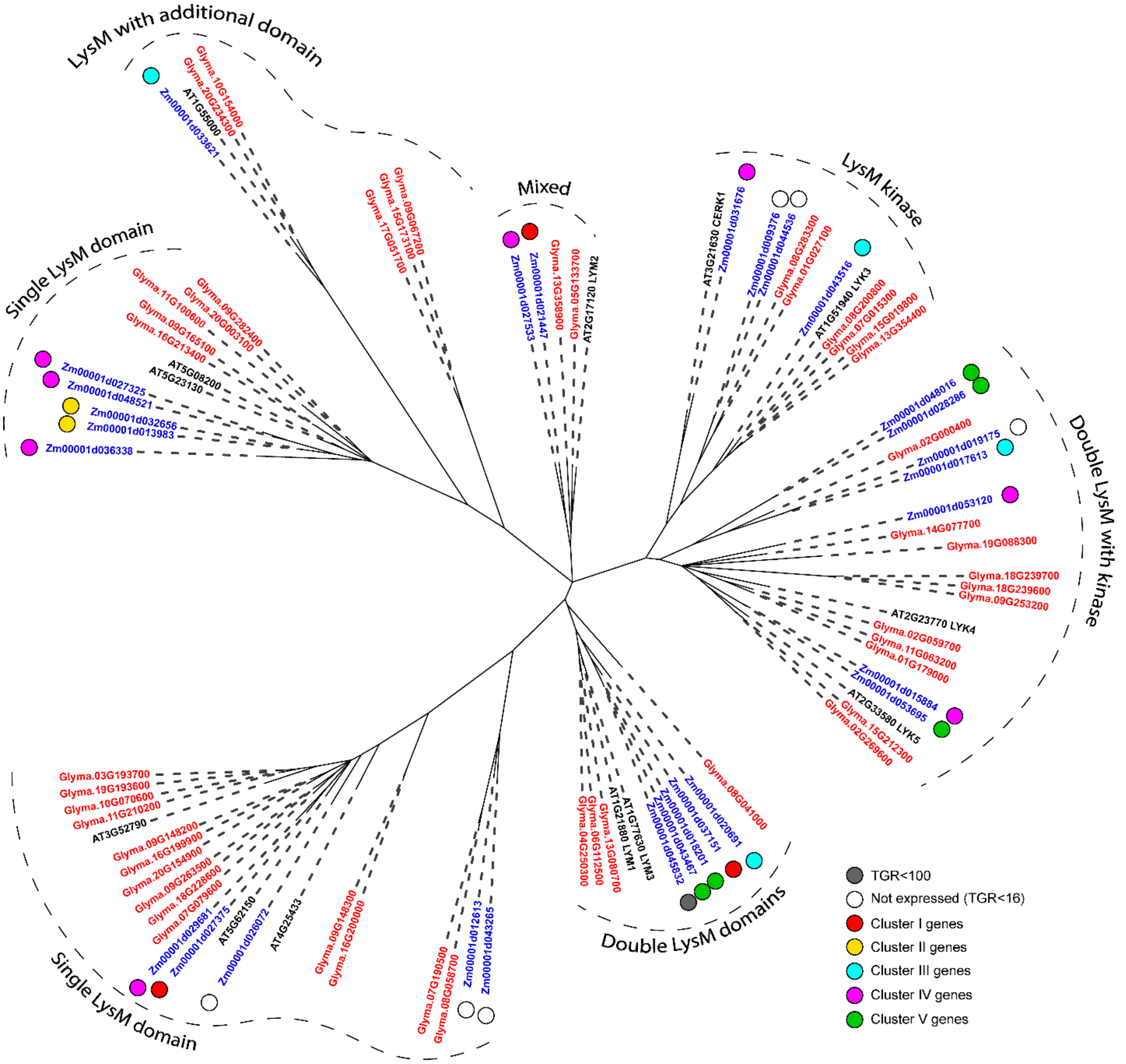

Figure S5. The phylogenetic dendrogram of LysM (PF01476) lectin family members of *A. thaliana* (gene names are given in black font), *Z. mays* (gene names are given in blue font) and *G. max* (gene names are given in red font). The different colored dots next to *Z. mays* gene names indicate genes with different expression patterns: white are genes with no expression (TGR value less than 16 in all samples) in studied zones of maize root; grey are genes with TGR value less than 100; red are genes from I cluster, the expression decreased from the zones of meristem and root cap to the late elongation; yellow are genes from II cluster, the expression peak was at the initiation of cell elongation; blue are genes from III cluster, they showed a high expression from the start to the late elongation; purple are genes from IV cluster, the expression increased in the root cap and also raised from the meristem zone to the late elongation; green are genes from V cluster, the expression raised to the late elongation. Only branches with the ultrafast bootstrap support values more than 90 are shown.

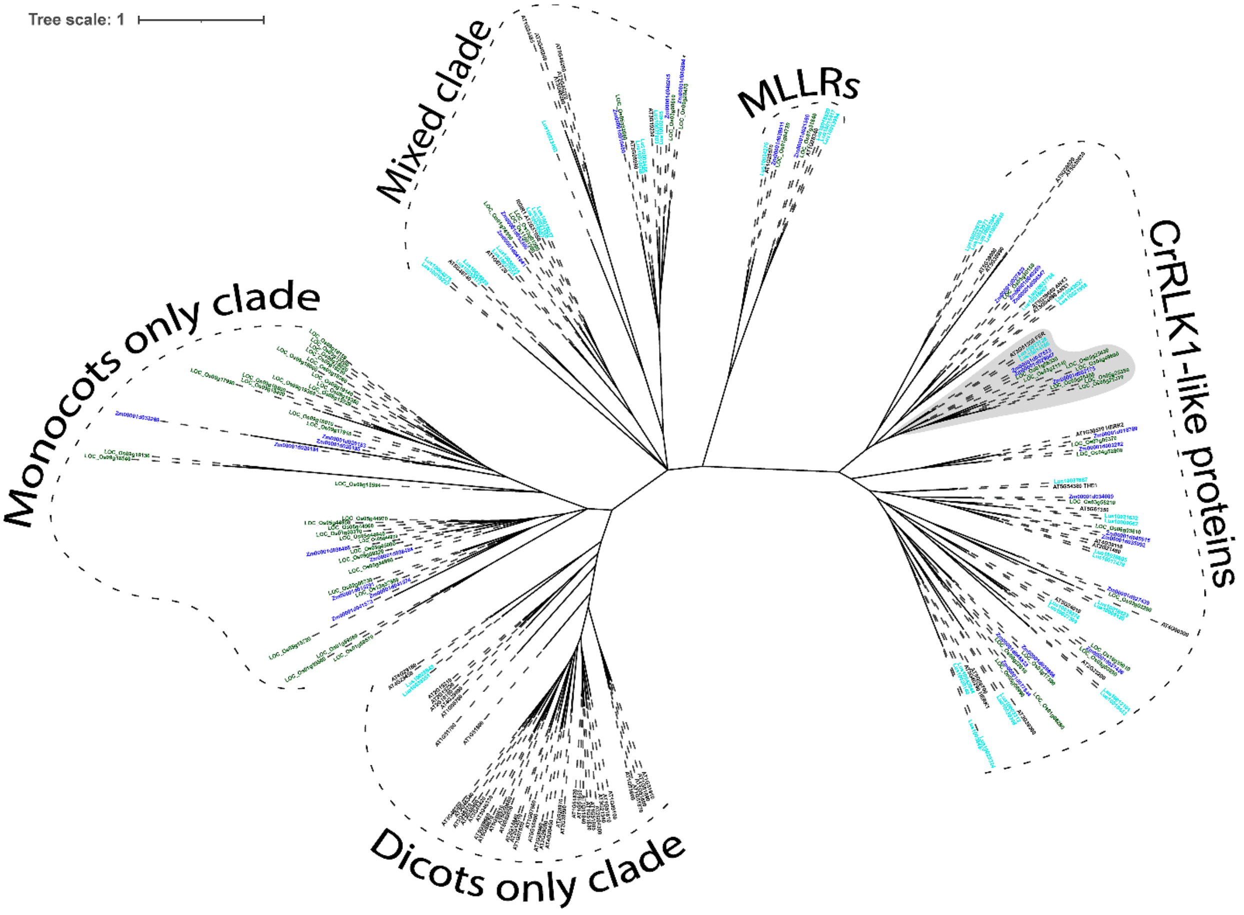

Figure S6. The phylogenetic dendrogram of malectin-like (PF12819) family members of *A. thaliana* (gene names are given in black font), *Z. mays* (gene names are given in blue font), *L. usitatissimum* (gene names are given in cyan font) and *O. sativa* (gene names are given in green font. Only branches with the ultrafast bootstrap support values more than 85 are shown. The subclade containing FER of *A. thaliana* and its homologues from other species is highlighted in gray.

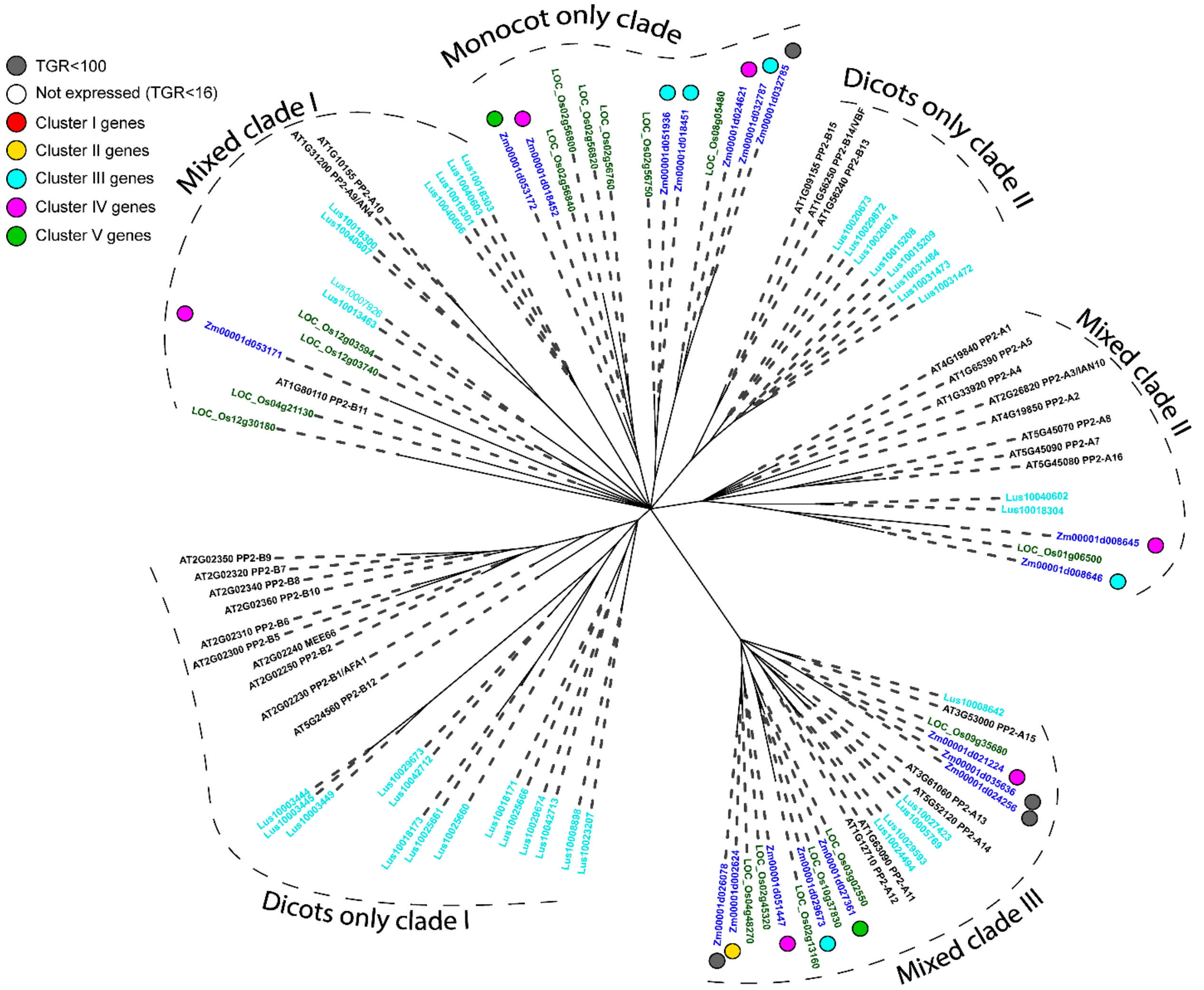

Figure S7. The phylogenetic dendrogram of Nictaba (PF14299) lectin family members of *A. thaliana* (gene names are given in black font), *Z. mays* (gene names are given in blue font), *O. sativa* (gene names are given in green font) and *L. usitatissimum* (gene names are given in cyan font). The different colored dots next to *Z. mays* gene names indicate genes with different expression patterns: white are genes with no expression (TGR value less than 16 in all samples) in studied zones of maize root; grey are genes with TGR value less than 100; red are genes from I cluster, the expression decreased from the zones of meristem and root cap to the late elongation; yellow are genes from II cluster, the expression peak was at the initiation of cell elongation; blue are genes from III cluster, they showed a high expression from the start to the late elongation; purple are genes from IV cluster, the expression increased in the root cap and also raised from the meristem zone to the late elongation; green are genes from V cluster, the expression raised to the late elongation. Only branches with the ultrafast bootstrap support values more than 90 are shown.
